# TBK1 mutation and its role in frontotemporal dementia and amyotrophic lateral sclerosis in Brazilian families

**DOI:** 10.1590/1980-5764-DN-2024-0227

**Published:** 2025-10-10

**Authors:** Rachel Leirner Argelazi, Santhiago Calvelo Graça, Pedro Vilaça Thomazoni, Carolina Braga Moura, Ana Carolina Gomes, Matheus Kohama Kormanski, Sephora Sabrina Candido de Almeida, Yngrid Dieguez Ferreira, Diogo Haddad Santos

**Affiliations:** 1 Faculdade de Ciências Médicas da Santa Casa de São Paulo, São Paulo SP, Brazil.; 2 Irmandade da Santa Casa de Misericórdia de São Paulo, São Paulo SP, Brazil.

**Keywords:** Frontotemporal Dementia, Amyotrophic Lateral Sclerosis, Mental Disorders, Epidemiology, Genetics, Demência Frontotemporal, Esclerose Lateral Amiotrófica, Transtornos Mentais, Epidemiologia, Genética

## Abstract

Frontotemporal dementia (FTD) and amyotrophic lateral sclerosis (ALS) are progressive neurodegenerative diseases with unclear etiology. Mutations in the TBK1 gene are associated with an increased risk of both FTD and ALS, presenting a diverse phenotype that includes the behavioral variant of FTD, primary progressive aphasia, and pure ALS. This mutation is rare, and to date, only one case report on TBK1-related clinical manifestations has been published in Brazil.

**Objective:**

To investigate the association between TBK1 gene mutations and the clinical manifestations of FTD and ALS in a Brazilian family, documenting the clinical history and disease progression of three first-degree relatives. Additionally, to conduct a literature review to better understand the impact of this mutation and its implications for neurological practice.

**Methods:**

Clinical data were collected from three patients in the same family who were receiving care at Dom Pedro II Geriatric Hospital and Central Hospital of the Irmandade da Santa Casa de Misericordia de São Paulo, including information on clinical symptoms, disease progression, and complementary exams — particularly genetic testing to detect and confirm the diagnosis. A detailed analysis of the existing literature on the disease was also conducted to better understand the implications of this mutation.

**Results:**

Three siblings affected by the TBK1 gene mutation were documented, with a unique family history suggesting that this genetic alteration has affected the lineage for several generations.

**Conclusion:**

Although rare, frontotemporal dementia with accompanying motor deficits is of significant relevance to neurologists due to its poor prognosis and the potential familial impact on descendants.

## RESUMO.

A demência frontotemporal (DFT) e a esclerose lateral amiotrófica (ELA) são doenças neurodegenerativas progressivas de etiológia ainda pouco compreendida. As mutações no gene TBK1 estão associadas a um risco aumentado para ambas as condições, apresentando um fenótipo diverso que inclui a variante comportamental da DFT, afasia progressiva primária e ELA pura. Trata-se de uma mutação rara, e, até o momento, apenas um relato de caso sobre manifestações clínicas relacionadas ao TBK1 foi publicado no Brasil.

**Objetivo:**

Investigar a associação entre mutações no gene TBK1 e as manifestações clínicas de DFT e ELA em uma família brasileira, documentando o histórico clínico e a progressão da doença em três parentes de primeiro grau. Além disso, realizar uma revisão da literatura para compreender melhor o impacto dessa mutação e suas implicações para a prática neurológica.

**Métodos:**

Foram coletados dados clínicos de três pacientes da mesma família atendidos no Hospital Geriátrico Dom Pedro II e no Hospital Central da Irmandade da Santa Casa de Misericórdia de São Paulo, incluindo informações sobre sintomas clínicos, progressão da doença e exames complementares — com destaque para testes genéticos com o objetivo de detectar e confirmar o diagnóstico. Uma análise detalhada da literatura existente sobre a doença também foi realizada para aprofundar o entendimento das implicações dessa mutação.

**Resultados:**

Foram documentados três irmãos com mutação no gene TBK1, com histórico familiar sugestivo de que essa alteração genética afeta a linhagem há várias gerações.

**Conclusão:**

Embora rara, a demência frontotemporal com comprometimento motor associado possui grande relevância para neurologistas, tanto por seu prognóstico reservado quanto pelo possível impacto familiar hereditário nas gerações subsequentes.

## INTRODUCTION

 Frontotemporal dementia (FTD) is a progressive neurodegenerative condition that leads to loss of autonomy and widespread physiological dysfunction, ultimately resulting in premature death. The main symptoms include behavioral and personality changes — such as disinhibition, loss of empathy, impaired judgment, and impulsivity — as well as speech-related alterations, including apraxia and aphasia. Unlike most forms of cognitive decline that primarily affect older adults, FTD often presents earlier in life and represents a significant cause of dementia in younger populations. Early recognition is crucial to enable appropriate treatment. 

 Regarding its etiology, numerous studies have aimed to elucidate its underlying mechanisms. In general, a genetic component is involved in its pathophysiology, with various genes implicated, such as microtubule-associated protein tau (MAPT), neighboring progranulin (GRN) gene, DNA-binding protein 43 (TDP-43), and hexanucleotide repeat expansions in the chromosome 9 open reading frame 72 (C9orf72) gene^1^. One gene of particular interest is the TBK1 (TANK-binding kinase 1) gene, which is associated with amyotrophic lateral sclerosis (ALS)^2^. 

 ALS is the primary disorder affecting motor neurons, with motor impairment as the main symptom. Patients with ALS may experience nasal and slurred speech, difficulty chewing or swallowing, and muscle contractions in the legs, arms, shoulders, or tongue, among other symptoms. Although ALS shares some clinical features with FTD, its etiology is multifactorial, and genetic factors play a minimal role in its pathophysiology^3^. However, TBK1 gene mutation is an exception, as its presence is associated with both ALS and FTD^2^. 

 The gene is located on chromosome 12, region 1, band 4, sub-band 2 (12q14.2), and encodes a protein of the same name. The TBK1 protein is involved in various biological processes, including autophagy, mitophagy, and components of innate immunity; its dysregulation is associated with dysfunctional mechanisms that can induce neuroinflammation^4^. In ALS, mutation of the gene impairs autophagy signaling, promoting protein accumulation and the formation of intracellular aggregates in motor neurons. As a result, a cytotoxic environment mediated by protein aggregation is established, leading to numerous alterations in neural homeostasis^2^. Ultimately, neuroinflammation ensues, leading to motor neuron degeneration and the clinical symptoms of the disease under study^5^. 

 Epidemiological data on this gene are scarce, but association have been reported with various other conditions beyond ALS and FTD, including certain thyroid cancers^6^ and cholangiocarcinoma^7^. A Belgian study identified the mutation of this gene as the third most frequent cause of clinical FTD in Belgium^8^. In Brazil, only one study has been conducted on this subject, emphasizing the need for further research to establish the frequency of this gene in the Brazilian population^9^. 

 Against this backdrop, the present study delves into a more in-depth investigation of a Brazilian group of patients who experienced rapid progression to frontotemporal dementia with motor involvement and delays in receiving a diagnosis of the aforementioned gene mutation. 

## METHODS

 Data from patients at the Dom Pedro II Geriatric Hospital, collected between 2014 to 2022, revealed a TBK1 Chr12:64.481.994 A>T variant in three Brazilians brothers. Informed Consent Forms were completed by the patients. In addition, to better understand the impact of this mutation on carriers, a thorough analysis of existing data in the PubMed literature was conducted. 

## RESULTS

### The older brother

 On April 22, 2021, the brothers GLS, 45 years of age, and JLS1, aged 43, presented to the neurology outpatient clinic of our institution. Both had a significant history of cognitive decline beginning around the age of 20, with progressive features and subsequent dependence on others for daily life activities within a few years. They were accompanied by a nursing assistant from Dom Pedro II Geriatric Hospital, GLS’s wife, and their aunt. 

 In 2021, GLS was 45 years old. He was born and raised in São Paulo, identified as mixed race, married, and the father of three children, as well as a grandfather. He had completed part of high school education and worked as a cleaning assistant. His wife, present at the consultation, reported a history of depressive episodes in his youth, along with tobacco and alcohol use, which he ceased in 2007. GLS’s clinical history revealed a complex progression of symptoms over the years. In 2013, he experienced a depressive episode characterized by social withdrawal, anhedonia, apathy, and insomnia. During this period, he also began talking to himself and to inanimate objects. Despite initiating treatment with Risperidone, there was no improvement. The following year, in 2014, after being dismissed from his job, his behavioral symptoms worsened, culminating in a suicide attempt. By 2015, he was under care at the Psychosocial Care Center (*Centro de Atenção Psicossocial* – CAPS) with a diagnosis of non-organic psychosis. He exhibited persistent apathy, avolition, reduced appetite, selective mutism, and disorganized thought and behavior. His condition deteriorated further, with the onset of suicidal ideation, significant weight loss, memory impairment, and temporal-spatial disorientation. In 2016, he developed persecutory delusions and auditory hallucinations. 

 In 2017, a molecular study for Huntington’s disease was conducted at Hospital São Paulo, with negative results for expanded alleles associated with the condition. Between 2017 and 2018, there was a rapid progression to severe cognitive and functional impairment, becoming fully dependent on basic activities of daily living, developing immobility syndrome, hand contractures, muscle stiffness, severely limited communication (reduced to incomprehensible sounds), and self-injurious behavior. Additionally, there was urinary and fecal incontinence, dysphagia, and dysarthria. In October 2018, Hospital Santa Casa collected cerebrospinal fluid and administered pulse therapy for suspected autoimmune encephalitis; however, no clinical improvement was observed. 

**Figure 1:**
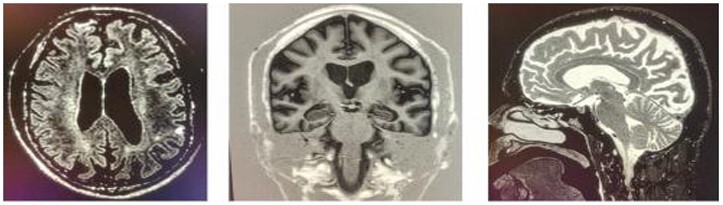
Brain magnetic resonance images of patient GLS, in which we can see cortical atrophy in the frontal lobe, as well as a reduction in global brain mass.

 Magnetic resonance imaging (MRI) findings across different periods revealed significant structural brain changes. In November 2017, mild to moderate brain volume loss and ventricular enlargement were observed. By May 2018, a diffuse volumetric reduction in brain tissue was noted, particularly in the parietal lobes. In February 2019, supratentorial brain atrophy was reported (Figure 1), predominantly in the occipital and parietal lobes, with corticosubcortical encephalomalacia/gliosis in the right frontal and left parietal lobes, likely from previous brain injuries. Cranial tomography in January 2016 showed no expansive lesions above or below the tentorium region. Cortical sulci, fissures, and liquid spaces were proportionate and only slightly prominent for his age at the time. However, by May 2020, tomography revealed pronounced widening of cerebral sulci and fissures, as well as cerebellar folia beyond expected parameters, along with compensatory ectasia of the upper brain ventricles, widened basal cisterns, and calcifications in the walls of the intracranial segments of the internal carotid arteries. An EEG conducted in November 2018 was within normal limits, showing no significant abnormalities. Laboratory tests conducted in 2019 for HIV, hepatitis B, hepatitis C, and syphilis yielded non-reactive results. 

### The middle brother

 Also present at the consultation on April 22, 2021, was GLS’s younger brother, JLS1, the middle child of three siblings. JLS1 was a single, mixed-race man, childless, a former security guard, aged 43, born and residing in São Paulo, with a history remarkably similar to that of his brother. According to family members, the patient had exhibited behavioral changes for the past four years. Until that time, the patient had been functional, involved in musical activities, and professionally active. He denied any history of hypertension, diabetes mellitus, smoking, or excessive alcohol consumption. However, in 2014, at the age of 36, he was dismissed from his job due to behavioral changes and aggressiveness in the workplace. Beginning in 2017, he began to socially isolate himself. At some unspecified point, he started exhibiting episodes of refusing personal care and food, neglecting household hygiene, screaming, and developing tongue bites. He progressed to limb tremors, dysarthria, cognitive decline, and eventually became completely dependent for activities of daily living. In December 2018, he was admitted to Hospital Dom Pedro. Since then, his condition has evolved into a state of persistent social isolation, with limited communication, episodes of psychomotor agitation and aggression, spending most of his time confined to a hospital bed. On physical examination, JLS1 followed simple commands but did not respond to complex commands and was unable to repeat or name items. He presented with apraxia and an oromandibular tic. 

**Figure 2:**
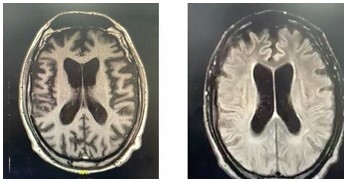
Brain Magnetic Resonance Images of the skull of patient JLS1, in which we can see global atrophy and predominance in bilateral frontotemporal regions and relative preservation of parieto-occipital regions.

 In January 2019, in pursuit of a diagnosis, JLS1 underwent cranial MRI, which revealed diffuse volumetric reduction, most prominent in the caudate nuclei, raising the possibility of Huntington’s disease as one of the main differential diagnoses. Rare foci of alteration in the white matter were also observed, suggesting gliosis associated with microangiopathy (Fazekas I). In a subsequent MRI, conducted in August 2019, global volumetric reduction of brain tissue was identified, especially in the caudate nuclei and, to a lesser extent, in the putamen (Figure 2). There was evidence of moderate volume loss in structures such as the hippocampal formations and entorhinal cortex, as well as subtle signs of microangiopathy/gliosis in the cerebral white matter. This set of clinical and radiological findings suggested a complex and progressive condition, possibly associated with Huntington’s disease or a related neurodegenerative process, necessitating detailed evaluation and continued clinical monitoring. 

### The younger brother

 As reported by family members during the April 2021 consultation, the clinical picture observed in the siblings was, unfortunately, common within the family. The third brother, the youngest of the three, JLS2, aged 36 at the time, lived in the back of his aunt’s house under poor hygiene conditions, struggled to maintain employment, and refused health care services. According to medical records, he had been documented as healthy three years earlier (2019). Additionally, it was reported that JLS2’s son had learning difficulties. The siblings’ father, paternal uncle, paternal grandmother, and a cousin had also experienced similar conditions. The father was reported to have begun showing symptoms around the age of 20 and passed away at the age of 46; the cousin began experiencing symptoms at age 25 and was hospitalized with a diagnosis of schizophrenia. In all cases, symptoms began around the age of 20 and progressed through the fourth and fifth decades of life, with initial features including depressed mood, social isolation, anhedonia, insomnia, and loss of social skills. These were accompanied by periods of psychomotor agitation and aggression. Over the following years, patients developed motor alterations, including executive deficits in the upper and lower limbs; immobility syndrome with hand contractures and stiffness; dysarthria; dysphagia; urinary and fecal incontinence; and significant memory loss. In the final stage of disease progression, occurring between the fourth and fifth decades of life, individuals presented with even more severe cognitive deficits, becoming able to follow only simple commands, fully dependent on assistance for basic daily activities, and confined to bed. 

### Family diagnosis

 In May 2022, a genetic test was conducted on GLS to investigate the familial etiology of the disease, and a variant in the TANK Binding Kinase 1 (TBK1) gene was identified. This variant, named Chr12:64,481,994 A>T (or c.965A>T – ENST00000331710), results in the substitution of the amino acid histidine with leucine at codon 322 (p.His322Leu). The position of histidine 322 is highly conserved across various biological species. However, computational analyses predicting pathogenicity indicate that this substitution to leucine may be neutral. This specific variant is found in heterozygosity in approximately 1 in every 120,000 individuals in population databases and has been reported in a variant repository. However, according to Freischmidt et al.^10^, haploinsufficiency of TBK1 is an established cause of familial frontotemporal dementia and/or amyotrophic lateral sclerosis 4 (OMIM #616439), which are neurodegenerative disorders characterized by cognitive impairment, behavioral abnormalities, speech apraxia, and upper and lower motor neuron dysfunction. While some TBK1 variants may be classified as variants of uncertain significance (VUS) under ACMG guidelines, this specific variant has been described in association with disease, supporting its pathogenicity. These conditions are progressive and genetically determined, following an autosomal dominant inheritance pattern. Thus, it is concluded that the clinical signs and symptoms presented by the family members had a genetic basis. 

### The end

 In November 2022, GLS arrived at our facility presenting with tachypnea, decreased oxygen saturation (70% in room air), subfebrile temperature, and excessive secretions. He had been on empirical antibiotic treatment since October. With the suspicion of bronchopneumonia, the medical team decided not to escalate antibiotics and prioritized comfort measures. 

 It was reported to the service that GLS and JLS1 had passed away, but the clinical course of the youngest brother, JLS2, and other family members remains unknown. 

## DISCUSSION

**Figure 3:**
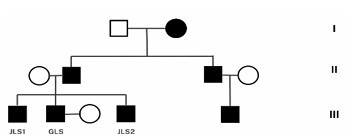
The genogram highlights the dominant inheritance pattern, characterized by the vertical transmission of the affected phenotype across generations and consistent manifestation of the disease in individuals caring the dominant allele.

 The current investigation on a Brazilian family with a mutation in the TBK1 gene (Chr12:64,481,994 A>T) resulting in FTD and ALS significantly contributes to the global understanding of the genetic bases of these neurodegenerative conditions. The discovery of three siblings affected by this genetic variation (Figure 3), along with a family history of similar neurological symptoms, underscores the importance of the genetic component in the etiology of FTD and ALS, corroborating previous studies that identified mutations in the TBK1 gene as a risk factor for the development of these diseases^2,11^. 

 The TBK1 gene encodes a crucial kinase involved in the immune response and signaling by phosphorylating target proteins associated with this system. Its main function is to phosphorylate and activate transcription factors, such as interferon regulatory factor 3 (IRF3), which, upon activation, translocates to the nucleus and induces the expression of IFN-β genes. This cytokine activates antiviral genes, modulates the organism’s immune response, and regulates intracellular vesicle trafficking. Furthermore, the protein encoded by this gene interacts with adapter proteins such as TANK, optineurin, and NAP1. TANK plays a role in activating the NF-κB pathway and the immune response, facilitating antiviral signaling through its interaction with TBK1 and other kinases. Optineurin is involved in autophagy, vesicle trafficking, and regulation of the immune response, known for its role in neuroprotection and prevention of neurodegenerative diseases. NAP1 is important for TBK1 activation in response to stress and inflammation signals^12^. 

 Pathogenic mutations in TBK1 are expected to impair autophagy mechanisms and crucial innate immunity signaling pathways. In relation to ALS and FTD, mutations cause dysfunction in autophagy, leading to the accumulation of misfolded proteins and damaged organelles in motor neurons, known as ribonucleoprotein inclusions. These inclusions are typically cleared by autophagy, and their accumulation contributes to the neurodegeneration observed in neurodegenerative diseases such as ALS and FTD. Additionally, dysfunction of the TBK1 gene can interfere with the DNA damage response, compromising the ability of motor neurons to adequately respond to cellular stress and increasing their vulnerability to degeneration. Finally, TBK1 alteration can lead to a dysregulated inflammatory response in the central nervous system, contributing to the neuroinflammation observed in ALS^13,14^. 

 The phenotypic heterogeneity observed in this family, ranging from cognitive deficits to motor difficulties, reflects the complexity and clinical variability associated with mutations in the TBK1 gene, as documented in the literature^15^. This phenotypic variability highlights the need for a careful and personalized clinical approach to the diagnosis and management of these patients. It also brings to light a complex reality within the Brazilian context, where multidisciplinary teams and genetic testing are not routinely available within the public health system^16^. 

 Regarding Brazilian cases published to date, reports remain scarce, which increases the relevance of the current work. One of the few cases described in 2022^9^ reinforces the importance of genetic research in identifying mutations in the TBK1 gene in populations previously underrepresented in this context. The inclusion of this case in the global panorama of research on FTD and ALS helps to fill gaps in knowledge regarding the geographical distribution and prevalence of these mutations and underscores the importance of considering genetic diversity in Latin American populations. This is the second case published in a scientific journal and the third described in the Brazilian population. 

 The classification of the genetic variation identified in this family, according to the guidelines of the American College of Medical Genetics (ACMG), highlights the challenges in clinical interpretation of genetic mutations and in the genotype-phenotype correlation. Computational analysis suggesting that the amino acid substitution may be neutral contrasts with the severity of the phenotypes observed, indicating that additional pathogenic mechanisms or modifying genetic or environmental factors may be involved^17^. 

 This study also emphasizes the importance of awareness and early recognition of the symptoms of FTD and ALS, particularly in individuals with a family history of these conditions. Early diagnosis and appropriate management are essential for improving the quality of life of patients and their families, as well as for providing valuable information for genetic counseling. The genetic complexity of ALS and its overlap with other neurodegenerative conditions, including FTD, reinforces the need for an integrated approach to understanding neurodegeneration, considering the interaction between different genetic mutations and their phenotypic effects^18^. 

 Furthermore, the discovery of a hexanucleotide repeat expansion GGGGCC in the non-coding region of the C9ORF72 gene^19^ as a cause of FTD and ALS linked to chromosome 9p reinforces the notion that genetic research continues to play a crucial role in elucidating the molecular basis of these complex diseases. This mutation, along with the TBK1 mutation discussed in the present study, exemplifies the genetic diversity underlying FTD and ALS and highlights the importance of exploring this diversity across different populations. 

 The detailed documentation of this family case in Brazil provides valuable insight into the genetic and clinical complexity of FTD and ALS associated with TBK1 gene mutation. In addition to reinforcing the need for further research to better understand the underlying pathogenic mechanisms, explore therapeutic options, and develop early intervention strategies, this report also underscores the importance of including diverse populations in genetic studies to capture the full spectrum of human genetic diversity and its effects on health and disease, especially in a mixed and heterogeneous population sch as the Brazilian one. 

## Data Availability

The datasets generated and/or analyzed during the current study are available from the corresponding author upon reasonable request.
